# Current Challenges in Volatile Organic Compounds Analysis as Potential Biomarkers of Cancer

**DOI:** 10.1155/2015/981458

**Published:** 2015-03-30

**Authors:** Kamila Schmidt, Ian Podmore

**Affiliations:** Biomedical Science Research Centre, School of Environment and Life Sciences, University of Salford, Manchester M5 4WT, UK

## Abstract

An early diagnosis and appropriate treatment are crucial in reducing mortality among people suffering from cancer. There is a lack of characteristic early clinical symptoms in most forms of cancer, which highlights the importance of investigating new methods for its early detection. One of the most promising methods is the analysis of volatile organic compounds (VOCs). VOCs are a diverse group of carbon-based chemicals that are present in exhaled breath and biofluids and may be collected from the headspace of these matrices. Different patterns of VOCs have been correlated with various diseases, cancer among them. Studies have also shown that cancer cells *in vitro* produce or consume specific VOCs that can serve as potential biomarkers that differentiate them from noncancerous cells. This review identifies the current challenges in the investigation of VOCs as potential cancer biomarkers, by the critical evaluation of available matrices for the *in vivo* and *in vitro* approaches in this field and by comparison of the main extraction and detection techniques that have been applied to date in this area of study. It also summarises complementary *in vivo*, *ex vivo*, and *in vitro* studies conducted to date in order to try to identify volatile biomarkers of cancer.

## 1. Introduction

Cancer is the second leading cause of death in the world. It has been estimated that there were 7.6 million fatal cases of cancer (13% of all deaths) and around 12.4 million new cancer cases in the year 2008 worldwide. Deaths from cancer are forecasted to continue to grow to over 13.1 million in 2030 [1]. The earlier the cancer is detected, the better the chances of the patient recovering are, as appropriate treatment can be applied in time. There are two components of efforts to detect cancer early: early diagnosis and screening. However, there is a lack of characteristic early clinical symptoms in most cancer types that could lead to early detection of the disease [2–5]. In addition, cancer diagnosis often requires many tests, some of which are invasive surgical procedures. Existing noninvasive methods often have limitations. For example, a new, noninvasive method of lung cancer screening, spiral computer tomography, which has been shown to detect cancer that is curable by surgery, is also accompanied by a risk of exposure to radiation, high false-positive rates, and the possibility of overdiagnosis [6]. This underlines the need for investigation of new methods for the early detection of cancer. In this search all “omics” approaches (genomics, proteomics, and metabolomics) have been applied [7–9]. One of the most promising metabolomic approaches is the analysis of volatile organic compounds (VOCs), which could potentially serve as a safe, noninvasive (at least for breath and some biofluid samples), and specific test for the early detection of different types of cancer.

VOCs are a diverse group of carbon-based chemicals that are classified on the basis of their retention time and boiling point (ranging from 50°C to 260°C) [10]. VOCs are emitted from the body in exhaled breath and are present in body specimens such as blood, urine, faeces, and sweat [11–14] and therefore may be collected from the headspace (HS) of these matrices, but also from the HS of cells* in vitro* [15]. Different patterns of VOCs have been correlated with various diseases and syndromes such as cancer [16], asthma [17], cystic fibrosis [18], diabetes [19], tuberculosis [20], chronic obstructive pulmonary disease [21], heart allograft rejection [22], and irritable bowel syndrome [13]. These correlations are based on the hypothesis that pathological processes, occurring as a consequence of disease, can generate new VOCs that the body does not produce during normal physiological processes and/or alter the concentrations of VOCs. These new VOCs, or VOCs that are produced in significantly higher or lower levels than normal, may therefore serve as biomarkers for the assessment or detection of disease.

This review firstly discusses sample matrices that were used in the studies of potential VOC biomarkers of cancer and critically evaluates* in vitro* and* in vivo* approaches applied in this field. The investigation of targeted VOCs only (rather than all the VOCs present in a sample) as candidate cancer biomarkers is also discussed. Next this paper reviews complementary* in vivo*,* ex vivo*, and* in vitro* studies conducted to date in order to find volatile biomarkers of cancer. Finally, the main extraction techniques and analytical techniques that have been applied to date in the area of the studies of potential volatile biomarkers of cancer are compared.

## 2. Available Approaches for VOCs Collection

In order to investigate VOCs as cancer biomarkers, analysis of the exhaled breath of patients with different types of cancer has become very popular in recent years [23, 24]. Alternative approaches include the HS analysis of cancer cells, tissues, or body fluids. All sample matrices have their advantages and disadvantages.

### 2.1. *In Vivo* VOCs Collection

#### 2.1.1. Breath Analysis

Studies have shown that chemical changes in blood due to the presence of cancer are echoed in an alteration of the composition of VOCs in the breath of patients [25, 26]. Therefore, it is hypothesised that abnormal VOCs produced by cancer cells are discharged via the blood stream into the endobronchial cavity and finally exhaled with breath [27].

Breath analysis, compared to blood and urine tests, is noninvasive and a sample may be easily collected at any point and in varying quantities, which makes it easy to repeat [28]. Furthermore, it does not require special storage conditions or any further work after collection. In addition, the breath matrix is a less complex mixture than urine or blood. There are approximately 200 VOCs present in a breath sample. However, they are not the same for each individual. Around 3500 different VOCs were detected in the breath of 50 people, and only 27 were found in the samples of all the subjects. Approximately half of these 3500 compounds are of possible endogenous and half of possible exogenous origin [29]. New volatile compounds are still being identified. Only compounds produced inside the body can be considered as biomarkers, which is problematic as the origin of most volatile metabolites is still unknown or remains the subject of speculation [27, 30]. The presence of both endo- and exogenous VOCs in exhaled air is one of the biggest limitations of breath analysis. Another is qualitative and quantitative interindividual and intraindividual variability. The majority of the detected VOCs were found only once in one particular individual [29] and the patterns of VOCs may change according to food consumption, smoking, gender, age, and so forth [31, 32].

There are different opinions about how detailed knowledge is required for a successful breath diagnostic test. Some argue that there is no need to know the origin of a volatile compound biomarker, as long as it can be used to distinguish disease from a healthy state [33, 34]. Others simultaneously measure exhaled and inspired air since the environmental contaminant VOCs may be incorrectly assigned as endogenous compounds [35]. Finally, the last approach requires knowledge about the metabolic pathway of the compound, as well as about normal concentration ranges of a compound in relation to interindividual variability, before including it into the predictive model of the disease [36].

Moreover, since the beginning of breath analysis in the 1970s [11] standardisation and reproducibility of the sample collection method have been an issue which has resulted in the variability of quantitative information [37, 38]. Standardisation is easier to achieve for serum or urine than for breath collection [37], which is a big advantage of these matrices. Furthermore, equipment for exhaled breath collection is relatively expensive and may thus not be easy to apply widely [23]. The importance (limitations and/or applications) of breath analysis has been described previously [30, 31, 37–42].

#### 2.1.2. Breath Analysis versus Body Fluids

Although VOCs detected in blood and urine are “in the body” analytes, it still does not mean they are of endogenous origin. Some inhaled VOCs may bind to or dissolve in blood [43] and be stored in body compartments and later excreted through urine [44]. In addition, it is not known which volatile compounds are produced or consumed by tumour cells as they may also be generated (or consumed) by noncancerous cells (such as surrounding tissue cells or other regions of the body) [45, 46], immune-competent cells [47], human symbiotic bacteria [48, 49], and infectious pathogens [50, 51]. Furthermore, VOC patterns differ between individuals because of uncontrolled variables such as genetic differences, environmental settings, diet, drug ingestion, and smoking [31, 32], which makes VOC analysis a challenge regardless of the matrix used. Nevertheless, there is growing evidence that VOCs that are potentially clinically relevant may be found in breath and other matrices. Dogs were reported to discriminate between patients with or without cancer by sniffing skin, blood, urine, or breath samples of cancer patients, which suggests that characteristic VOC signatures of cancer exist [52–57]. Sensor mice were also trained to distinguish mice with experimentally-induced cancer from mice without it [58].

Blood was used as a matrix for VOC collection in a number of studies of lung cancer [26, 59], childhood forms of cancer [60], and liver cancer [61]. The disadvantages of blood as a matrix include invasiveness, and careful handling and further work after collection as temperature and pH changes can alter VOC profile [37, 62]. Moreover, there are difficulties in the collection of arterial blood. When there is a necessity to collect many of such samples, breath analysis would be a better alternative, especially as it closely mirrors the arterial concentrations of metabolites [23]. In theory, the composition of volatile compounds in breath is related to the composition of these compounds in blood [23, 26]. This needs to be addressed in studies comparing VOC composition in blood and breath samples. Such an investigation concerning cancer was performed by Deng et al. [26]. The study showed that 23 VOCs found in blood were also present in the exhaled breath of lung cancer patients. Therefore, there are characteristic compounds which identify cancer presence. Among these 23, hexanal and heptanal were detected only in cancerous blood and breath samples and were not found in controls. However, more study is required to compare VOC patterns in both matrices, where ideally the blood and breath samples from the same patient would be investigated.

Many studies have also investigated volatile biomarkers in urine samples of patients with various cancers such as breast [63], gastroesophageal [64], lung [65], leukaemia, colorectal, lymphoma [44], childhood leukaemia [60], and bladder cancer [66]. In addition to its noninvasive nature and availability in large volumes, urine as a matrix for VOC analysis also has an advantage over other biofluids in that analytes are concentrated by the kidney before being excreted from the body. In addition, when compared to blood, the use of urine usually results in better detection limits as matrix effects may interfere with the release of the VOCs into HS in blood sampling [67]. On the other hand, VOCs in urine may be affected by the drugs administered to a patient, and therefore the metabolic products of particular changes must be known as well as determining their effect on the VOCs produced [66].

### 2.2. *In Vitro* VOCs Collection

The investigation of VOCs produced by cancerous cells in the microenvironment as the source of biomarkers should hypothetically help with the dilemma of their origin, as advantages of* in vitro* studies over other matrices include easier control of experimental variables and more easily interpreted results, due to the absence of factors such as gender, age, and interindividual variation (with the exception of primary cell cultures) [68]. They also offer lower cost and better reproducibility. However, this matrix still does not guarantee that the collected VOCs are of endogenous origin. They may not be produced by cancer cells themselves and may instead come from other sources such as culture vessels, extraction devices, and the sampling environment [69, 70].

The cell metabolome is comprised of the endometabolome, which is represented by all metabolites inside the cell, and the exometabolome, which is made up of all metabolites present in the extracellular cell culture medium. The profile of these metabolites in the surrounding medium depends on the uptake and extraction of the compounds by the cells and reflects their metabolic activity via their response to experimental variables.* In vitro* studies aiming to find potential volatile markers of cancer essentially apply the extracellular metabolite investigative approach. Endometabolomic studies require cell disruption, and then concentration of the extracted compounds (mainly with the use of evaporation). VOCs could be easily lost during these steps [68].

A number of studies have been performed to investigate potential VOC cancer biomarkers* in vitro* in different types of cancer and using different techniques, and in all of them there were differences observed in the composition of volatile metabolites produced by cancer and normal cells [69–81].

However, some studies found differences in VOC levels, or VOCs produced, between not only different cell lines of the same cancer (showing that their metabolic pathways are different) but also the same cell line [15, 75, 79–82]. While the first observation may be explained by genetic and phenotypic differences and the fact that each cell line is representative of only a small part of a primary tumour, the reasons for the second are unclear [15, 80]. It may be due to the cell line being subcultured a different number of times. The study of Sponring et al. [72] showed the possibility of a change of released volatile metabolites with increasing passage number. Cells should not be subcultured for a long period of time to ensure they have not mutated, as mutation could cause them to no longer reflect the properties of the tumour of origin. The fact that there were significant experimental differences in many studies between the cell cultures that had been subcultured a low number of times, compared to those that had been subcultured a high number of times, and the fact that there were studies conducted on cross contaminated cell lines make a compelling case for the use of certified cell lines with defined passage numbers [83].

In the cell/tissue HS analysis of VOCs there are also differences in the techniques used, and a lack of standardisation and normalisation of the data, even when the same technique is used, which may influence variations in VOC patterns between different studies. The aspects to be considered (apart from the technique used) (Table 1) in terms of* in vitro* studies of VOCs include the analysis of different matrices, the use of different cell culture media, the period of cell cultivation, the different cell density, the different cell controls used, the different statistical methods used, and finally the differing methodology.

Length of incubation periods and differing types of culture (in monolayer, matrix immobilized cultures, or 3D cultures) as well as supplementation of cell culture medium have been shown to have an influence on the composition of the VOCs in the samples [79, 81, 84–86]. Drug addition also has been shown to change the pattern of VOCs produced by A549 cells* in vitro*, highlighting the possibility of finding biomarkers of apoptosis and necrosis induced by drugs [87].

The main matrices analysed to study VOCs generated by cells are (i) HS of the cell-free culture medium of a target cell and (ii) HS of the medium still containing the cells. The HS of cell lysate (preconcentrated supernatant of the lysed cells) is another matrix employed, but has only been used in a few studies, solely for the determination of targeted VOCs produced by cancer cells treated with drugs (Table 1).

There are some substantial differences in terms of the extraction procedure details for the main two matrices. For example, analysis of culture media with cells usually takes place at 37°C (physiological conditions), while analysis of media only may employ a higher temperature. Also, the efficiency of analysis of media only samples can be improved by the addition of salts or by a change of pH, while such changes are not possible when cells are present. On the other hand, the analysis of media with cells ensures that no VOCs are lost during storage. Finally, the vessel used for cell culture is of great importance. Some researchers use glass vials as they have very limited release of volatile chemicals (other materials such as standard plastic flasks for cell culture release plasticizers generating additional peaks) [69, 88].

### 2.3. *In Vitro* versus* In Vivo* Analysis

A recent review by Kalluri et al. [89] makes a case that the studies* in vivo* and* in vitro*, investigating VOCs as potential biomarkers of cancer, have poor correlations (specifically studies of lung cancer and exhaled breath as a sample matrix). They postulate that the overlap between VOCs found in the exhaled breath of lung cancer patients and compounds produced by lung cancer cells* in vitro* (approximately one-quarter being common to both matrices) is not sufficient at the moment for* in vitro* culture to be a good model for the VOCs present in exhaled breath. The authors propose that it could be due to cell cultivation in hyperoxic conditions (atmospheric oxygen concentration) emphasising it as a potential limitation of the* in vitro* studies performed to date. Tumours have been shown to grow in hypoxic (oxygen depleted) or anoxic (oxygen absent) conditions as opposed to normal tissues [90]. Cellular oxidative stress would lead to the production of different VOCs by cells in comparison to hyperoxic cell culture conditions. Studies comparing the patterns of VOCs present in the HS of cells cultured in hyperoxic and hypoxic conditions are needed to address this potential limitation of* in vitro* approach.

However, another issue related to cell culture conditions could also result in the different VOCs present in the HS of cell culture and samples taken from the patient. Standard 2D cell culture conditions may have a great impact on the cell metabolic behaviour, thereby losing accuracy when looking for biomarkers when compared to 3D culture that better mimics the growth of the tumour [81].

The poor correlation between* in vivo* and* in vitro* studies may also arise from exogenous VOCs being included in the predictive models of cancer [30], different extraction and detection techniques used in different studies, different experimental design, and in general a relatively lower number of* in vitro* studies performed to date, in comparison to the VOC studies of breath samples and biofluids. In addition, studies which show that the VOC patterns do not change after tumour removal imply that some VOCs may be biomarkers of the risk of cancer developing, rather than being indicative of the presence of a tumour (see Section 2.4 for further discussion). Also it is important to remember that there is very little known about the complexity of the transmission mechanisms of the VOCs produced by tumour cells in the body and found in breath or biofluids. An excellent review of lung cancer VOC studies, which describes possible biological pathways of lung cancer VOCs identified from different matrices, shows that this is a main challenge to date for cancer VOC analysis [27]. Therefore, the composition of VOCs found in the samples from the patients and VOCs detected in the HS of cultured cells cannot be expected to be the same. However the studies that have been performed to date in order to find potential volatile biomarkers of cancer show that the VOCs common to all matrices exist, regardless of the potential limitations of the* in vivo* and* in vitro* approaches discussed above.

### 2.4. Complementary Studies* In Vivo*,* Ex Vivo*, and* In Vitro*

Without doubt, there is a need for a simultaneous investigation of the correlation of the VOC pattern in exhaled breath (and other sample types) collected from a patient and an* in vitro* and/or* ex vivo* analysis of the VOCs produced by the cancer cells or emitted from the cancer tissues (ideally of the same patient). This approach eliminates analytical technique and, in the case of the samples coming from the same patient, factors such as gender, age, and interindividual variation as the sources of possible differences in VOC patterns between* in vivo*,* in vitro*, and* ex vivo* samples.

Some studies already have been conducted specifically in order to simultaneously compare VOCs produced by cancer cells* in vitro* and* ex vivo* to the ones found in breath from the patient.

The study of Chen et al. [78] aimed to compare VOCs produced by four types of primary lung cancer cells to VOCs found in cancer breath samples. In this study, 11 VOCs were found in breath samples and chosen for principal component analysis in order to discriminate cancer patients from healthy controls, and two compounds were shared with lung cancer cells excised from the patients (namely, isoprene and undecane) [78].

Another study compared volatile metabolites determined in a culture medium of lung cancer cell line A549 to the VOC composition in the HS of urine of mice implanted with these cells. There were seven VOCs found at significantly higher levels in both sample types when compared to normal cancer cell lines (dimethyl succinate, 2-pentanone, phenol, 2-methylpyrazine, 2-hexanone, 2-butanone, and acetophenone) [79].

The study performed by Buszewski et al. [28] involved quantitative VOC measurement in the HS of healthy and lung cancer tissues and comparison of these results to the ones obtained from the breath samples of the healthy individuals and lung cancer patients. 27 VOCs were detected in the air above cancerous tissues, cutting down the number of potential biomarkers that need to be considered when breath samples are analysed. 22 of the same compounds (mainly alcohols, aldehydes, ketones, and aromatic and aliphatic hydrocarbons) were found in the breath samples, just as in the HS of lung tissues. Quantitative analysis of VOCs emitted by lung cancer tissues showed higher levels of ethanol, acetone, acetonitrile, 1-propanol, 2-propanol, carbon disulfide, dimethyl sulfide, 2-butanone, and 2-pentanone when compared to control lung tissues. The same compounds were detected in increased concentrations in the breath samples of patients suffering from lung cancer when compared to healthy controls. Some of them were detected in the HS of cancer cells in previous studies (Table 2).

The exhaled breath of lung cancer patients was compared not only to the breath of healthy controls, but also to the compounds detected in the HS of lung tissues (cancerous and healthy), again in the recent study by Filipiak et al. [91]. They detected 39 VOCs in both types of samples: tissue specimens, and exhaled breath (with different occurrence ranging from 8 to 100%). Over half of the detected compounds were previously reported in the HS of cancer cells* in vitro* in different studies (Table 2). Although approximately half of the VOCs in the breath samples had negative alveolar gradient (alveolar gradient: abundance in breath minus abundance in the air), suggesting their exogenous origin, these findings show common VOCs in all three sample types. Out of 39 detected, they found 30 VOCs at higher concentrations in cancerous lung tissue, when compared to the healthy tissue controls. Six were elevated at the chosen level of significance: ethanol, pyridine, 4-methylheptane, acetaldehyde, n-octane in the HS of lung cancer tissues, and n-hexanone in the HS of healthy tissues. Ethanol and octane were also found at significantly higher levels in the breath of lung cancer patients. What is more, these compounds were previously detected in the HS of lung cancer cells* in vitro*. Acetaldehyde and 4-methylheptane were also found in the HS of cultured cancer cells. Other VOCs found in higher levels in the cancerous lung tissue (but not at significant levels) such as 2-methyl-1-pentene, 4-methyloctane, 2,4-dimethylheptane, hexane, and acetic acid were also previously detected in the HS of different cancer cell lines (Table 2).

Poli et al. [92, 93] in their unique study measured VOC concentrations in the breath of lung cancer patients before and a month and three years after the excision of a tumour. In the study, they analysed 12 VOCs that were found in higher concentrations in the breath of cancer patients than in the breath of healthy controls before the surgery. They compared the concentrations of these analytes to the VOC levels found in cancerous and healthy lung tissue collected during the surgery. Collection and storage issues allowed for analysis only of aromatic hydrocarbons in the tissue specimens. Six aromatic VOCs were found to be common to the exhaled breath and tissue samples (benzene, ethylbenzene, trimethylbenzene, toluene, styrene, and xylenes). Their levels (except for xylenes) were significantly higher in cancerous tissue than in healthy tissue. Ethylbenzene, xylenes, and styrene were compounds detected in the HS of lung cancer cells in previous studies (Table 2). Interestingly, no differences in the levels of 11 of the VOCs (isoprene being the exception) were found between the breath collected before and one month after the tumour removal. Similar outcomes were obtained by Phillips et al. [94] who did not observe any changes in the VOC profiles in the breath of most lung cancer patients before and after surgery. In Poli et al.'s study, three years after surgery, the levels of some of these compounds had changed (decreased for isoprene and benzene, increased for pentane, toluene, and ethyl benzene) [92].

The findings of both Poli et al.'s and Phillips et al.'s studies imply that changes in the VOC patterns are not biomarkers of lung cancer presence but rather are epiphenomenon of the disease development. In fact Phillips et al. [94, 95] proposed an upstream hypothesis which may explain these results as opposed to a downstream hypothesis. In the latter, the presence of the disease causes the altered patterns of VOCs in breath samples. In Phillips et al.'s pathophysiologic model, altered VOC profiles in the breath of the person suffering from lung cancer and the presence of the disease itself are somewhat independent. A person carrying high-risk genotypes due to exposure to carcinogens will have induced activity of the enzymes catabolising VOCs. The patterns of the volatile metabolites may therefore be altered before the appearance of the tumour. Excision of the tumour does not eliminate the induced activity of the enzymes. However, the fact that common VOCs were found in the breath, the HS of lung tissues of cancer patients, and the HS of cancer cells grown* in vitro*, in addition to the fact that the levels of some compounds changed after a longer period following an operation to remove a tumour, implies that at least some of the VOCs are produced by the tumour* per se* and may not be attributable to genetic predispositions. Further studies are required to confirm any of these hypotheses.

### 2.5. Analysis of Targeted VOCs

A different approach to the issue of the possible exogenous origin of proposed VOC biomarkers focuses on the detection of aldehydes [12, 59, 65, 96] or hydrocarbons [97–99] only as markers of cancer. Studies proved that oxidative stress is one of the main sources of developing cancer via the overproduction of reactive oxygen species and nitrogen species resulting in mutations [100]. Some aldehydes are known to be related to oxidative stress as they are products of lipid peroxidation, but the exact mechanism of their presence in breath and body fluids is not known [27, 101, 102]. The same mechanism underlies the emission of saturated hydrocarbons in the body. They are products of lipid peroxidation of polyunsaturated fatty acids (PUFA) [27]. This process does not involve branched hydrocarbons as there are no branched polyunsaturated fatty acids in the body [103], nor does it appear to involve methylated alkanes as there is not enough data to support their origin from lipid peroxidation [104]. As aldehydes are highly reactive and can easily decompose or react while the sample is prepared for analysis or storage, a chemical derivatization has been introduced [96]. One of the most common derivatization methods for aldehyde determination is the reaction of aliphatic aldehydes with PFBHA O-(2,3,4,5,6-pentafluorophenyl)methylhydroxylamine hydrochloride to produce stable oximes [105]. Different studies that employed different techniques of extraction demonstrated this as an effective method for aldehyde analysis in various matrices [12, 60, 106–108].

Higher concentrations of straight C3–C9 aldehydes [32, 65, 96, 106, 108], as well as some unbranched hydrocarbons [28, 93, 97, 99], were identified among VOCs in cancerous breath, blood, and urine matrices in many studies. What is more, some of these VOCs are the analytes that have been found common to the HS of cancer cells* in vitro* and exhaled breath of lung cancer patients [89].

## 3. Techniques of Extraction and Detection for Investigation of VOCs as Potential Cancer Biomarkers

### 3.1. Extraction Techniques

Concentrations of most of the VOCs present in biological matrices are low: in the nmol^−1^–pmol^−1^ (ppb–ppt) range in exhaled human breath and in the *μ*mol^−1^–nmol^−1^ (ppm–ppb) range in blood and urine [12, 35, 37, 60]. In addition, VOCs are analytes of interest to be extracted from complex mixtures. Therefore, prior to the assay, a preconcentration step is required, which is the most labour-intensive part of the analysis and is the primary source of errors influencing the reliability and accuracy of analysis [109]. Increased reproducibility and elimination of interfering compounds can be achieved by minimising the number of steps. The ideal properties of a sample-preparation device include simplicity, high extraction capacity and selectivity, efficiency, speed, possible automation and miniaturization, compatibility with a range of separation and detection methods, and safety in use for the operator and environment [110, 111]. Microextraction methods employ some of these features the best, when compared to the traditional sampling techniques of liquid-liquid extraction and solid-phase extraction. Solid-phase microextraction (SPME) in particular became very popular due to its simplicity and lack of solvent use and the fact that it has been automated and is compatible with GC-MS and LC-MS [112].

**Figure 1:**
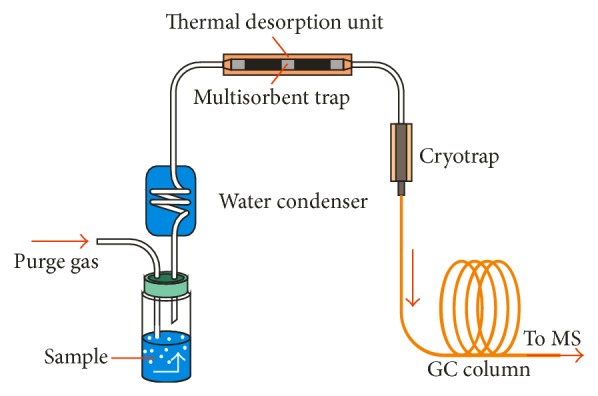
Diagram of analysis with online purge and trap-gas chromatography-mass spectrometry (PT-GC-MS).

Purge and trap (PT) and solid phase microextraction (SPME) are the two main extraction techniques used to date for the collection of VOCs in both* in vivo* and* in vitro* studies of potential cancer biomarkers. In PT (also called dynamic headspace extraction) the gas sample is purged through the sorbent trap by an inert gas and the VOCs are retained on the surface of the trap (Figure 1). Next they are thermally desorbed with the use of an online thermal desorption (TD) device or extracted with small amounts of solvents (liquid desorption: LD). Sorbent traps are adsorption materials contained in a small tube. The most commonly used sorbent traps for the analysis of VOCs employ charcoal (e.g., Carbotrap) or porous polymer (e.g., Tenax) as a trapping material with varying degrees of selectivity. TD may cause degradation reactions of sensitive compounds and column degradation, as some sorbents have a high affinity to water [113]. There are various techniques for water removal in PT such as the use of drying gas, a water condenser, or an additional adsorption trap [114]. LD is a milder technique, so it does not cause degradation of sensitive VOCs; however it is less sensitive [113]. In studies of potential VOC cancer biomarkers only TD-PT has been employed (with cryofocusing to enhance resolution) (e.g., [35, 80]).

**Figure 2:**
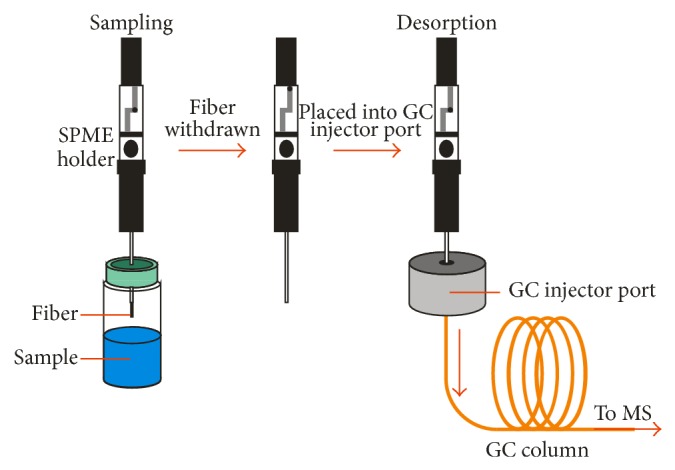
Diagram of analysis with solid phase microextraction-gas chromatography-mass spectrometry (SPME-GC-MS).

SPME is an extraction technique where an extraction phase is dispersed on a fine rod made of fused silica, Stableflex, or metal alloy. The SPME device consists of two parts: the holder and, contained in it, the fiber assembly. There are two versions of the SPME holder: one for manual use and one for use with autosamplers or with a high performance liquid chromatography-SPME (HPLC-SPME) interface. The fiber unit consists of a fiber core attached via a hub to a stainless steel guiding rod, which is contained in a hollowed needle that pierces a septum. The fiber is withdrawn from this needle when sampling and the needle is removed when not in use (Figure 2). The fiber core is 1 or 2 cm long and is coated with stationary phase. The fiber is immersed in the liquid sample in the case of direct immersion (DI-SPME) or suspended in the HS above the sample (HS-SPME). During extraction, sample molecules preferentially partition from matrix to stationary phase as a result of adsorption or absorption. In the adsorption process the analytes remain on the surface of the trapping material due to chemical bonding. In the absorption process, the analytes are dissolved into the bulk of a liquid phase (e.g., PDMS) [115]. After sampling, the analytes are thermally desorbed in the injector port with no use of solvents (Figure 2).

There are several commercially available SPME fibers for sampling a wide range of compounds that employ four polymers as stationary phases: divinylbenzene (DVB), polydimethylsiloxane (PDMS), polyacrylate (PA), and polyethyleneglycol (PEG). They are used on their own as a coat (available in different thicknesses) or in combination blended with carboxen (CAR). The coatings differ by polarity (polar, bipolar, and nonpolar) and extraction mechanism (absorbent or adsorbent). The choice of fiber coating depends on the polarity of analytes and their molecular weight. Previous analyses of VOCs as biomarkers of cancer has been performed in most cases with the use of a 75 *μ*m CAR/PDMS coating regardless of the type of matrix tested. Its use is justified, as the fiber was initially developed for the extraction of volatile and small compounds [116].

In comparison, a sorbent trap is an exhaustive extraction technique, due to chemical reactions between the stationary phase and the analytes, whereas SPME is a nonexhaustive (passive) equilibrium technique where the amounts of VOCs extracted are controlled by the series of distribution constants between the gaseous, liquid, and coating phases. Sorbent trapping is based on an adsorption process. SPME, depending on the fiber used, is an absorption technique or utilizes absorption and adsorption properties simultaneously. Sorbent trapping is a three-step process (extraction of the analytes to the solid sorbent, desorption, and cold focusing), whereas SPME is more simple in use (sorption of analytes onto the fiber then desorption) [115]. The simplicity of SPME facilitates the development of normalised methods and standardisation [31]. SPME-GC-MS does not require an additional device connected to the gas chromatograph such as a cryotrap or a water removal device. On the other hand, as SPME methodology is limited by the commercially available fibres, its sensitivity is also limited. Sorbent traps may employ additional preconcentration, such as using higher volumes of the trapping material to enhance sensitivity [117]. The sensitivity of SPME is not as dependent on sample volume as sorbent traps; the limits of detection of the latter technique get better with a larger volume of sample [41]. SPME use is limited when large sample volumes are analysed. For example, the use of sorbent traps showed an order of magnitude lower limit of detection (LOD) than SPME for isoprene in human breath, when the same, 8 L breath samples were analysed [118]. LODs obtained in studies analysing VOCs as potential cancer biomarkers showed that PT extraction technique yielded better sensitivity (low ngl^−1^ in full scan mode) than SPME (*μ*gl^−1^ in full scan mode) [80, 119].

As selection of the appropriate fiber coating for analytes is a critical stage in the SPME methodology development, there are new fiber coatings under development with higher capacity and selectivity, which would enhance sensitivity such as molecularly imprinted polymers, multiwall carbon nanotubes, sol-gel technology, and polymeric ionic liquids (reviewed in [120, 121]).

Other variations of SPME techniques such as stir bar sorptive extraction (SBSE), solid phase microextraction membrane, (or thin-film microextraction), and needle trap device have been successfully used for the collection of VOCs and so may be used in cancer studies in the future (reviewed in [111, 120]). Needle trap device has been already used by Mochalski et al. [122] for analysis of VOCs in the HS of liver cancer cell line. Another microextraction method, single drop microextraction, was also introduced for the HS analysis of VOCs in cancerous blood. The technique is simple and rapid, uses trace amounts of solvents (2 *μ*L), and is less costly than SPME [106]. Barash et al. [73, 123] used Ultra II SKC passive (no purge) diffusion badges for the preconcentration of VOCs from the HS of the cell culture media. In this type of sampler sorbent traps serve as adsorption material, and extraction is based, as in SPME, on the equilibrium principles [124]. Offline sorbent trapping was also used by Amal et al. (with the use of TD) [71]. Finally, cryoconcentration was also used prior to analysis in a study, in order to investigate VOCs produced by leukaemia cell line. The VOCs were quantified in trace levels (low ppb). Separation of the analytes was achieved here by the use of multicolumn GC [125].

### 3.2. Detection Techniques

The main detection techniques that have been employed in VOC cancer biomarker studies are GC-MS, proton transfer reaction-mass spectrometry (PTR-MS), selected ion flow tube-mass spectrometry (SIFT-MS), and gas sensors (electronic noses) (Table 3). Sampling and analytical techniques for the analysis of VOCs in biological samples are summarised in the recent review by Zhang et al. [111] and for breath analysis specifically in the reviews by Di Francesco et al. and Kim et al. [31, 40]. Advances and/or applications in gas sensor technology in breath analysis have been recently described in a number of reviews [126–132].

GC-MS is the most commonly used analytical technique for the investigation of potential VOC cancer biomarkers, due to its sensitivity and reliability in analyte identification. It gives the most detailed analytical information and identifies analytes with the most certainty, when compared to PTR-MS. The identification of VOCs with the use of PTR-MS can be tentative only as it is not possible to discriminate between compounds with the same molecular weight [74, 88, 133]. On the other hand, PTR-MS is the most sensitive method of all, with the limit of detection for aromatic hydrocarbons in low-ppb levels [134], or even as low as a few ppt [135]. It has been demonstrated to be more sensitive than GC-MS measurement by a factor of ~20 [134]. GC-MS was shown to have sensitivity for VOC analysis at the ppb and low ppt levels but it needs a further preconcentration step [96, 117]. SIFT-MS allows for the measurement of trace gases at sub-ppb levels [136], but it is also reliable in the identification of compounds [134]. The advantage of PTR-MS and SIFT-MS over GC-MS is that they do not require a preconcentration step and can work in online (real time) mode. Therefore they are better techniques for the quantification of VOCs, as they provide instant quantification of all the analytes in the sample [133, 136] (Table 3). In comparison, SPME-GC-MS measures analytes semiquantitatively, as it involves competitive absorption of the compounds on the fiber [115]. GC-MS instruments are also more expensive. Nevertheless, instruments for all the techniques are not easy to use in clinical settings in terms of portability or transport [133]. Although the easily transportable SIFT (TransSIFT) and PTR (PTR-QMS 300) instruments have been introduced commercially [136, 137], their small sizes compromise their sensitivity.

Another detection technique, ion mobility spectrometry (IMS), is not very common yet in the studies of VOCs as potential cancer biomarkers, but already has shown promising results. The first study which applied IMS for the analysis of VOCs in the exhaled breath of lung cancer patients and healthy subjects was performed by Westhoff et al. [138]. Discriminant analysis employing 23 VOC peaks identified individuals with or without a tumour with 100% accuracy. In another study, the detection of different VOC concentrations in the breath of cancer patients using IMS allowed for discrimination between different histological subtypes of lung cancer [139]. The IMS detector is characterised by low selectivity. Therefore, complex mixtures are analysed with the use of a preseparation technique such as multicapillary column (MCC) or GC [140, 141]. Mainly IMS coupled with MCC has been used for breath analysis in the studies performed to date [138–140, 142]. The advantages of MCC-IMS include very fast analysis (500 s for the breath sample), no need for preconcentration, and online analysis. In contrast to other analytical techniques, the use of MCC-IMS allows for the detection of all the analytes in a breath sample through their separation by retention time, mobility, and concentration and by creating a 3D visualisation of each compound in the chromatogram [138]. Although it does not allow for the identification of the analytes, IMS is a comparatively cheap detection technique with a potential for miniaturisation and is therefore one of the most promising, next to electronic noses, candidates to be used in a clinical setting [140].

Compared to mass spectrometric methods, the use of electronic noses does not require skilled personnel and is less time consuming [143]. These features, as well as the potential miniaturisation of such devices [144], make them ideal potential diagnostic tools to be used by general practitioners or even as devices for personal use. There have been several types of electronic noses used in the studies of VOCs in cancer [33, 73, 143, 145–147]. They are designed to recognise VOC patterns emitted from the analysed samples, but not to identify these VOCs [145]. Generally, electronic noses have not been designed to quantify analyte intensity [148]. However, construction of calibration curves allows for the semiquantitative detection of VOCs [149]. Quantification of VOCs with the use of an electronic nose has not been performed in any studies of cancer.

In terms of breath testing, such sensor systems could be cheap, rapid, and simple to use when they have been tailored for a specific use [31]. However, electronic noses are highly sensitive to moisture and relatively less sensitive (1–5 ppb) [150] and their effectiveness needs more validation studies as they have shown poor linearity and reproducibility [151]. Nevertheless, electronic noses constitute a very promising research area in the analysis of VOCs as potential cancer biomarkers. For example, a novel combination of a GC separation system and metal oxide sensor device has already shown very good accuracy in diagnosing bladder cancer [147]. Quartz microbalance gas sensors also demonstrated very good accuracy in differentiation between lung cancer patients and healthy controls [152]. Finally nanomaterial-based chemiresistors have shown the ability to distinguish not only between the breath of patients suffering from different cancer types [143], but also between early and advanced stages of lung cancer, between the types of lung cancer, and between malignant and benign pulmonary nodules [153].

## 4. Conclusions and Future Directions 

Researchers take different approaches when looking for the potential biomarkers of cancer. The discussion starts with the issue of whether to choose an* in vivo* or* in vitro* system for study. Obviously the aim is to apply differential VOCs of cancer to a device that will enable the detection of cancer in the patient with 100% certainty, ideally noninvasively, as the less invasive a procedure is, the cheaper and more simple it will be to conduct. Whether it is going to be breath, blood, urine, or any other sample coming from the patient, at this stage, none of these matrices is ideal for looking for potential volatile biomarkers. The main reason is the uncertain origin of the detected VOCs, as their patterns may depend not only on the presence of the disease, but also on the long list of other variables such as genetic and environmental factors, age, gender, and so forth.

Therefore, it seems obvious to complement these studies with an investigation of the VOC profiles produced by tumours at the microcellular level, where an explanation of the presence of a compound in the chromatogram is more straightforward. The studies on cells are of great informative value about the biochemistry of tumours. However, with the* in vitro* approach, there are also some uncertainties arising. The main one is that there is little known about the complexity of VOCs metabolic pathways, between the VOC being produced by the tumour cells and its presence (or absence) in the sample from the patient. Nevertheless,* in vitro* studies are valuable tools in advancing the aim of cancer diagnosis.

Ideally, research should be directed to comparing VOC patterns in the HS of cancer cells or tissues of one particular patient with the compounds detected in breath, urine, and/or blood of the same patient. Also the selection of controls is crucial, in order to eliminate as many variables as possible. Without doubt, more studies are needed for the comparison of VOCs produced by tumour cells to the ones found in breath or biofluids, as well as for comparison of VOC patterns generated by many cell lines and primary tumour samples in order to profile as many cells as possible, so that an attempt can be made to find the common VOCs for particular types of cancer.

Each of the five types of analytical techniques that found application in the studies of VOCs has its advantages and disadvantages. Although it is more likely that a future tool to be used in the clinic will be an electronic sensor device, due to its cheaper cost, however, gas sensors still have poor sensitivities. Therefore other analytical techniques may be researched further. Consequently, for research purposes, it seems to be ideal to use the methods in complement.

Studies of the “scent of cancer” are really elegant in the simplicity of the idea; however there are still limitations of applying this idea clinically regardless of the technique used. At the moment certainty that any VOC is a biomarker of cancer is far from straightforward. Analysis of breath and other matrices investigating potential biomarkers of cancer is still in its infancy. Evidently large-scale screening studies are first required in order to describe normal profiles of VOCs in all matrices being studied. Knowledge about VOC concentration ranges for a normal, nondiseased state and validation studies using larger populations in relation to all forms of cancer will further evaluate the promising results of the existing studies of these diseases. And here surely the path to the use of VOCs as “smellprints” of cancer in the clinic lies in using information gleaned from a variety of different approaches in complement.

## Figures and Tables

**Table 1:** Analytical technique used, cancer cell lines studied, and type of matrix and control used in *in vitro* studies aiming to investigate VOCs as potential cancer biomarkers. DNTD: dynamic needle trap device; ESI: electrospray ionisation; GC-MS: gas chromatography-mass spectrometry; MC: multicolumn; Mm: metastatic melanoma cell; ns: not specified; NSCLC: non-small-cell lung cancer; p: preconcentration; PT: purge and trap; PTR-MS: proton transfer reaction-mass spectrometry; RPG: radial growth cell; SCLC: small-cell lung cancer; SIFT-MS: selected ion flow tube-mass spectrometry; SPME: solid phase microextraction; VPG: vertical growth cell.

Analytical technique used	Cancer type	Cell lines studied	Control	Type of matrix	Reference
SPME-GC-MS	Lung cancer	A549	OUS11 and WI-38 VA 13	Cell-free culture medium	[79]

SPME-GC-MS	Skin cancer	**RPG:** M35, WM3211, and Sbcl2 **VPG:** WM115 and WM983A **Mm:** WM983B and WM1158	FOM136, FOM191, and pure medium	Cell-free culture medium	[70]

SPME-GC-MS	Lung cancer	A549, SK-MEM-1, and NCIH 446	BEAS2B	Cell-free culture medium	[76]

SPME-GC-MS	Colon cancer	SW1116 and SW480	NCM460, pure medium	Culture medium with cells	[69]

SPME-GC-MS	Lung cancer	Primary lung cancer cells	Primary normal cells (human lung cells, lipocytes, osteogenic cells, and rat taste bud cells)	Cell-free culture medium	[78]

SPME-GC-MS	Lung cancer	A549	Pure medium	Culture medium with cells	[87]

Nanosensors (quartz microbalances), SPME-GC-MS	Melanoma, synovial sarcoma, and thyroid cancer	Primary cells	Pure medium	Culture medium with cells	[154]

Ultra II SKC-GC-MS, nanosensors (gold nanoparticles)	Lung cancer	**NSCLC:** A549, Calu-3, H1650, H4006, H1435, H820, and H1975	Pure medium	Culture medium with cells	[123]

Ultra II SKC-GC-MS, nanosensors (gold nanoparticles)	Lung cancer	**NSCLC:** A549, Calu-3, H1650, H4006, H1435, H820, H1975, H2009, HCC95, HCC15, H226, and NE18 **SCLC:** H774, H69, H187, and H526	IBE, pure medium	Culture medium with cells	[73]

ORBOTM 420 Tenax TA sorption tubes-GC-MS, nanosensors (gold nanoparticles; single walled carbon nanotubes)	Liver cancer	MHCC97-H, MHCC97-L, HepG2, SMMC-7721, and BEL-7402	L-02	Culture medium with cells	[71]

PT-GC-MS	Lung cancer	Calu-1	Pure medium	Culture medium with cells	[15]

PT-GC-MS	Lung cancer	NCI-H2087	Pure medium	Culture medium with cells	[72]

PT-GC-MS	Lung cancer	A549	HBEpC, hFB, and pure medium	Culture medium with cells	[80]

DNTD-GC-MS	Liver cancer	HepG2	Pure medium	Culture medium with cells	[122]

pMC-GC-MS (p: cryogenic)	Leukaemia	HL60	Pure medium	Culture medium with cells	[125]

SIFT-MS	Breast cancer	MCF-7 and MCF-7Adr	ns	Cell lysate	[155]

p-SIFT-MS (p: distillation)	Breast, leukaemia, cervical, and prostate cancer	MCF-7, MCF-7Adr, HeLa S3, K562, LNCaP, and DU-145	Solid residue left after centrifugation	Cell lysate	[156]

p-SIFT-MS	Breast cancer	MCF-7 and MCF-7Adr	Solid residue left after centrifugation	Cell lysate	[157]

SIFT-MS	Lung cancer	CALU1	NL20, pure medium	Medium with cells	[81]

PTR-MS	Lung cancer	A549 and EPLC	hTERT-RPE1, BEAS2B, and pure medium	Medium with cells	[74]

SIFT-MS	Lung cancer	Calu1 and SK-MEM-1	Pure medium	Medium with cells	[82]

SIFT-MS	Lung cancer	Calu-1	NL20, 35FL121 Tel+, and pure medium	Medium with cells	[75]

Online (ESI)MS	Breast cancer	T47D, SKBR-3, and MDA-MB-231	HMLE	Cell-free culture medium	[77]

**Table 2:** Volatile organic compounds detected in both the exhaled breath of lung cancer patients and the HS of lung cancer tissues in studies that simultaneously investigated VOCs *ex vivo* and *in vivo*. Only VOCs that have also been previously detected in the HS of cancer cells *in vitro* in other studies are listed.

Class	Volatile organic compound	Reference	*In vitro* study reference
Alkanes	Pentane	[28]	[72]
	Hexane	[28, 91]	[72]
	Octane	[91]	[80]

Branched alkanes	2-Methylpentane	[28, 91]	[72]
	3-Methylpentane	[28]	[72]
	2,3,4-Trimethylpentane	[91]	[72]
	4-Methyloctane	[91]	[15, 72]

Alkenes	2-Methyl-1-pentene	[91]	[80]
	2,4-Dimethyl-1-heptene	[91]	[72, 79, 80]

Alcohols	Ethanol	[28, 91]	[79, 80, 82]
	1-Propanol	[28]	[72]

Aldehydes	Acetaldehyde	[28, 91]	[15, 72, 74, 75, 81, 82, 125]
	Acrolein	[91]	[15]
	Hexanal	[91]	[15, 72, 74, 80, 122, 125]
	3-Methylbutanal	[91]	[72, 122]
	2-Methylpropanal	[91]	[15, 72, 80, 122]
	2-Methylbutanal	[91]	[72, 122]
	Benzaldehyde	[28, 91]	[15, 69, 70, 79, 122]

Ketones	Acetone	[28, 91]	[70, 80]
	2-Butanone	[28, 91]	[15, 72, 79]
	2-Pentanone	[28, 91]	[79, 80, 122]
	2-Hexanone	[91]	[79]
	6-Methyl-5-hepten-2-one	[91]	[70]

Carboxylic acids	Acetic acid	[91]	[82, 123]

Ethers	Diethyl ether	[28]	[79]

Pyrroles	Pyrrole	[91]	[79, 80]

Nitriles	Acetonitrile	[28, 91]	[15, 79, 122]

Aromatics	o-Xylene	[92]	[79, 123]
	p-Xylene	[28, 92]	[123]
	Ethylbenzene	[28, 92]	[123]
	Styrene	[92]	[79, 123]

**Table 3:** Main characteristics of analytical techniques used in the studies of VOCs as potential cancer biomarkers. GC-MS: gas chromatography-mass spectrometry; IMS: ion mobility spectrometry; MCC: multicapillary column; PTR-MS: proton transfer reaction-mass spectrometry; SIFT-MS: selected ion flow tube-mass spectrometry.

Analytical technique	Sensitivity	Quantification	Mode	Compound identification
GC-MS	Sub-ppb-low ppt^1^ [96, 117]	Semiquantitative	Offline	Reliable
PTR-MS	Low ppb-low ppt [134, 135]	Absolute	Real-time	Tentative
SIFT-MS	Sub-ppb-low ppb [136]	Absolute	Real-time	Reliable
MCC-IMS	ppb-ppt [140]	Absolute	Real-time	Tentative
e-noses	Low ppb [150]	Semiquantitative	Real-time	—

^1^With preconcentration.
